# Protein Kinase C δ (PKCδ) Attenuates Bleomycin Induced Pulmonary Fibrosis via Inhibiting NF-κB Signaling Pathway

**DOI:** 10.3389/fphys.2020.00367

**Published:** 2020-04-22

**Authors:** Jun Wang, Lei Sun, Yunjuan Nie, Shixin Duan, Tao Zhang, Weiwei Wang, Richard D. Ye, Shangwei Hou, Feng Qian

**Affiliations:** ^1^Engineering Research Center of Cell & Therapeutic Antibody, Ministry of Education, School of Pharmacy, Shanghai Jiao Tong University, Shanghai, China; ^2^Department of Basic Medicine, Wuxi School of Medicine, Jiangnan University, Wuxi, China; ^3^College of Pharmacy and Chemistry, Dali University, Dali, China; ^4^School of Life and Health Sciences, The Chinese University of Hong Kong, Shenzhen, China; ^5^Hongqiao International Institute of Medicine, Tongren Hospital, Shanghai Jiao Tong University School of Medicine, Shanghai, China; ^6^Anhui Province Key Laboratory of Translational Cancer Research, Bengbu Medical College, Bengbu, China

**Keywords:** PKC δ, pulmonary fibrosis, inflammation, macrophage, NF-κB signal pathway

## Abstract

Idiopathic pulmonary fibrosis (IPF) is a chronic, progressive and lethal interstitial lung disease characterized by consistent pulmonary inflammation. Although protein kinase C delta (PKCδ) is involved in broad scope cellular response, the role of PKCδ in IPF is complicated and has not been fully defined yet. Here, we reported that PKCδ deficiency (PKCδ^–/–^) aggravated bleomycin (BLM)-induced pulmonary fibrosis and inflammation. Upon challenge with BLM, the pulmonary capillary permeability, immune cell infiltration, inflammatory cytokine production, and collagen deposition were enhanced in PKCδ^–/–^ mice compared to that in PKCδ^+/+^ mice. In response to poly(I:C) stimulation, PKCδ deficient macrophages displayed an increased production of IL-1β, IL-6, TNF-α, and IL-33, which were associated with an enhanced NF-κB activation. Furthermore, we found that PKCδ could directly bind to and phosphorylate A20, an inhibitory protein of NF-κB signal. These results suggested that PKCδ may inhibit the NF-κB signaling pathway via enhancing the stability and activity of A20, which in turn attenuates pulmonary fibrosis, suggesting that PKCδ is a promising target for treating pulmonary fibrosis.

## Introduction

Idiopathic pulmonary fibrosis (IPF) is a progressive, devastating, and lethal interstitial lung disease. It has a prevalence of 7∼10 per 100,000 people worldwide and a mean survival of only 3∼4 years since diagnosis (Dempsey, 2006). Characterized by inflammation, fibroblast accumulation, and extracellular matrix deposition, pulmonary fibrosis eventually leads to the disruption of lung architecture that hinders blood gas exchange (Nie et al., 2017). Since the etiology and mechanism of IPF have not been fully unveiled (Desai et al., 2018) and current therapies have limited efficacy (Fujimoto et al., 2015), it is vital that new drug targets are to be identified as treatment options for the management and resolution of IPF.

Although the etiology of pulmonary fibrosis is complicated and unclear, inflammation is definitively involved in pathogenesis of IPF (Bringardner et al., 2008). Damaged tissue releases various inflammatory stimuli from the nucleus or cytosol, such as high mobility group box 1(HMGB1), DNA, RNA and heat shock proteins, which function as danger associated molecular patterns to trigger the sterile inflammation response (Mack, 2018). Among them, RNA is an important endogenous ligand for TLR3 that activates NF-κB signal pathway and double-stranded RNA released from damaged or death cells upregulates the expression of inflammatory mediators to enhance inflammation response (Kariko et al., 2004; Cavassani et al., 2008; Nelson et al., 2015; Li et al., 2017; Zhan et al., 2017). Poly(I:C), a mimic of dsRNA, is commonly used to study the role of RNA in immune response (Zhan et al., 2017). Pulmonary fibrosis has been considered as the result of wound repair and tissue remodeling. Epithelial injury leads to chronic inflammation and activation of inflammatory cells, such as macrophages and neutrophils, which release harmful reactive oxygen species, cytokines, and growth factors that regulate the proliferation and activation of fibroblasts and ultimately result in pulmonary fibrosis (Desai et al., 2018). Bleomycin (BLM) administration is the most widely used for inducing lung fibrosis in animal models, which triggers DNA strand scission in alveolar epithelial cells and subsequently induces cells damage or death (Della Latta et al., 2015). Double-stranded RNA released from damaged epithelial cells can activate NF-κB signal pathway to trigger inflammatory response and enhance pro-inflammatory cytokine expression (Nelson et al., 2015). Chronic and excessive inflammation triggers the continual unrestrained growth of fibroblasts in the formation of pulmonary fibrosis. Hence, the immune response and inflammatory environment play an important role in IPF (Wynn and Vannella, 2016).

PKCδ is the first identified member of the novel PKC subfamily, which is ubiquitously expressed in mammalian cells, including macrophages (Zhao et al., 2012). Tyrosine phosphorylation is an important way to regulate PKCδ activity. Hiroaki Konishi et al. have proved that Tyr311 is the predominant modification site compared with Tyr332 and Tyr512. Through the tyrosine phosphorylation detection *in vitro* and kinetic analysis, they demonstrated that the Tyr311 phosphorylation enhances the PKCδ basal enzymatic activity and elevates its maximal velocity in the presence of diacylglycerol. The mutation of Tyr311 to phenylalanine prevents an increase in this maximal activity (Konishi et al., 2001). In addition, several other groups have also demonstrated the important effect of the Tyr311 phosphorylation on PKCδ activity (Kikkawa et al., 2002; Hall et al., 2007; Nakashima et al., 2008). Hence, the Tyr311 phosphorylation can be used as a marker for the research of PKCδ activation. The PKCδ activation plays a critical role in many cellular response such as cell growth, differentiation, apoptosis, and phagocytosis. However, the role of PKCδ in macrophage activation and pulmonary is still controversial. PKCδ deficiency enhances the expression of IL-6 and TNF-α in macrophages and increases the IL-6 production in spleen tissue after infection of *Listeria monocytogenes*, which suggests that PKCδ can attenuate inflammatory response and the macrophages activation (Anita Schwegmann et al., 2007). During *Mycobacterium tuberculosis-*induced lung injury, PKCδ inhibits the expression of proinflammatory cytokines IL-1β, IL-6, and TNF-α by using PKCδ deficient mice and a PKCδ specific inhibitor (Parihar et al., 2018). In contrast, it was also reported that PKCδ is required for NF-κB activation and IL-8 expression in fibroblasts and epithelial cells in response to TNF-α treatment (Li et al., 2009; Lee et al., 2018). Hence, the role of PKCδ in inflammatory response is controversial and needed to be further investigated.

Alveolar macrophages are important immune cells present in the lung and play a key role in pulmonary diseases (Qian et al., 2015; Huang et al., 2019). Macrophages can promote fibroproliferation and lead to uncontrolled wound repair via secretion of various proinflammatory cytokines, fibrotic mediators and growth factors, such as TNF-α, IL-1β, CCL-18, IL-33, and TGF-β (Li et al., 2014; Nabe, 2014; Theoharides et al., 2015; Wynn and Vannella, 2016). BLM administration induces IL-33 production in murine pulmonary macrophages, which recruits and activates immune cells and finally promotes pulmonary fibrosis (Cayrol and Girard, 2014; Li et al., 2014; Xia et al., 2015). The NF-κB signal pathway is important for the regulation of inflammatory cytokine production (Wertz et al., 2004) which can be down-regulated by the A20 deubiquitinase, also known as tumor necrosis factor α-induced protein 3 (TNFAIP3) (Das et al., 2018). However, it is still unknown whether and how PKCδ modulates NF-κB signal activation in pulmonary fibrosis and TLR3-mediated signal pathway.

In this study, we used PKCδ deficient mice to determine the effect of PKCδ on BLM-induced pulmonary fibrosis. We found that PKCδ deficiency remarkably enhances BLM-induced inflammation and pulmonary fibrosis. Moreover, PKCδ deficiency enhances the proinflammatory cytokine expression in lung tissues and poly(I:C)-stimulated macrophages. In addition, PKCδ attenuates NF-κB activation through inducing phosphorylation and stability of A20. Our results indicated that PKCδ inhibits BLM-induced pulmonary fibrosis and is a promising drug target for IPF.

## Materials and Methods

### Mice

The original PKCδ knockout (PKCδ^–/–^) mice in C57BL/6 × 129 were kindly provided by Dr. Robert Messing (Chou et al., 2004) (Ernest Gallo Clinic and Research Center, University of California, San Francisco), these were backcrossed for 10 generations with Balb/c mice. The age- and sex- matched wild-type littermate controls were used for the experiments. Mice were housed in specific pathogen free conditions at the Laboratory Animal Center of Shanghai Jiao Tong University. The procedures were approved by the Institutional Animal Care and Use Committee at Shanghai Jiao Tong University.

### Bleomycin (BLM)-Induced Pulmonary Fibrosis Mouse Model

The male PKCδ^+/+^ or PKCδ^–/–^ mice (6–8 weeks) were administered BLM (BioTang, Beijing, China) dissolved in saline via a single intratracheal instillation under anesthesia at a dose of 1.6 U/kg body weight to induce pulmonary fibrosis (Nie et al., 2017). Control groups were administered with an equal volume of sterile saline. Mice were sacrificed at days 3, 7, 14, and 21 after BLM administration when the bronchoalveolar lavage fluid (BALF) and lung tissue were collected for further analysis. This included the number of infiltrating lymphocytes, total protein concentration in BALF, histology of lung tissue, as well as the level of cytokine expression and collagen content in lung tissues.

### Immunohistochemistry (IHC)

The lung tissue specimens were taken from six healthy donors (2 female, 47 and 35 years old; 4 male, 51, 53, 68, and 54 years old) and six recipients (3 female, 55, 49 and 52 years old; 3 male, 59, 74, and 48 years old) with pulmonary fibrosis during lung transplantation in Wuxi People’s Hospital (Jiangsu, China), using procedures approved by the Institutional Review Board of Wuxi People’s Hospital. Sections (5 μm) were cut from the paraffin-embedded blocks prepared from the human lung tissues. Washed the slides with the specific reagents in the following order: xylene, two times, 5 min each; 100% ethanol, two times, 5 min each; 95% ethanol, two times, 5 min each; 80% ethanol, once, 5 min; 70% ethanol, once, 5 min; 50% ethanol, once, 5 min; ddH_2_O, two times, 5 min each. In order to quench endogenous peroxidase, the slides were incubated in 3% H_2_O_2_ in distilled water for 5∼10 min. Following being rinsed with PBS three times, slides were incubated with 3% normal serum in PBS for 1 h to block non-specific binding. The slides were then incubated with p-PKC-delta antibody overnight at 4°C. Following being rinsed with PBS three times, slides were incubated with HRP-goat anti-rabbit polyclonal secondary antibody (1:200) for 1 h. Following being rinsed with PBS, the slides were covered with chromogen of final developmental DAB. Stained and differentiated slides in hematoxylin. After the dehydration, transparency and mounting of the slides, images of the tissues sections were captured by digital microscope.

### Lung Histology

On days 3, 7, 14, and 21 post-BLM treatment, the lungs from mice in each treatment group were collected. The right lungs were frozen until further use, while the left lung was collected after inflating with 1 ml of 4% paraformaldehyde (Sangon, Shanghai, China) under constant pressure and placed in 4% paraformaldehyde. The left lung tissues were then embedded in paraffin blocks and cut into 5 μm sections for hematoxylin and eosin (H&E) staining or Masson trichrome staining to observe the inflammatory cell infiltration and collagen deposition respectively (NanJing Jiancheng Bioengineering Institute, Nanjing, China).

### Lung Homogenization and Analysis

Hydroxyproline is an indicator of the level of collagen present in a sample. The hydroxyproline content was assayed in lung hydrolyzate according to the manufacturer’s protocol for the hydroxyproline assay kit (NanJing Jiancheng Bioengineering Institute, Nanjing, China). The absorbance of the colored product was measured at 550 nm using a microplate reader to evaluate collagen deposition. In addition, myeloperoxidase (MPO) activity in lung tissue was assessed using tetramethylbenzidine (TMB), as previously described (Qian et al., 2012).

### Bronchoalveolar Lavage

Mice were sacrificed on days 3 and 7 after treatment with saline or BLM. Following blood collection by cardiac puncture, the trachea was exposed and intubated with a polyethylene catheter. Lungs were lavaged three times with 0.6 ml PBS (PH 7.4). All resulting BALF was collected and centrifuged at 500 g for 5 min. The supernatant was used to detect total protein concentration using a BCA Protein Assay Kit (Beyotime, Shanghai, China) and the pellet was resuspended to determine the number of infiltrating cells using a hemocytometer (He et al., 2017).

### Isolation of Total RNA and Quantitative PCR (QPCR)

Frozen right lung lobes were homogenized with the TissueLyser system (Qiagen). Total cellular RNA in lung tissue homogenates or bone marrow derived macrophages (BMDM) as well as Raw264.7 cells were extracted by Trizol reagent (Thermo Fisher Scientific) (Wu et al., 2018). The cDNA was prepared by ReverTra Ace qPCR RT Kit (Toyobo, Japan) and amplified by real time PCR in an Applied Biosystems PCR instrument with target genes primer sets: α-SMA (forward 5′-GACGCTGAAGTATCCGATA GAACACG-3′, reverse 5′-CACCATCTC-CAGAGTC-CAGCAC AAT-3′), fibronectin (forward 5′-TCTGGGAAATGGAAA- AGGGGAATGG-3′, reverse 5′-CACTGAAGCAGGTTTCC TCGGTTGT-3′), IL-33 (forward 5′-GATGGGAAGAAG- CTGATGGTG-3′, reverse 5′-TTGTGAAGGACGAAGAAG GC-3′), TNF-α (forward-5′-GGCAGG-TCTACTTTGGAGTC ATTGC-3′, reverse 5′-ACATTCGAGGCTCCA-GTGAATTC GG-3′), IL1-β (forward5′-AGGACATGAGCACCTTCTTTT CC-3′, reverse5′-ACGT-CACACACCAGCAGGTTA-3′), IL-6 (forward5′-TCGGAGGCTTAATTACACATGTTC-3′, rever-se 5′-CATACAATCAGAATTGCCATTGC-3′). Relative gene expression levels was measured using the 2^–ΔΔCt^ method and was normalized to the GAPDH mRNA level.

### Preparation of Bone Marrow-Derived Macrophages (BMDM)

Femoral and tibia bone marrow was isolated from both PKCδ^+/+^ and PKCδ^–/–^ mice as previously described (Qian et al., 2019). The primary bone marrow cells were washed with PBS (PH = 7.4) and cultured in Dulbecco’s modified Eagle’s medium (DMEM, Thermo Fisher Scientific) with 10% fetal bovine serum (FBS Beyotime, Shanghai, China), 1% penicillin/streptomycin (Thermo Fisher Scientific), and 10 ng/ml M-CSF (Peprotech, Rocky Hill, CT, United States) for 6 days. These BMDMs were then stimulated by poly(I:C) (Invivogen, French, 100 μg/ml) before the cells were lysed in Trizol or RIPA Lysis buffer for RNA and protein expression analysis.

### Western Blotting (WB)

Three days after BLM administration, the right lungs were collected and lysed by RIPA Lysis buffer (Beyotime, Shanghai, China). The tissue lysate was analyzed by immunoblotting using an antibody against mouse IL-33. BMDMs were plated in six-well plates at 1 × 10^6^ cells per well overnight and challenged with 100 μg/ml poly(I:C) or PBS for different lengths of time. After platting THP1 cells in six-well plates at 1 × 10^6^ cells per well, the cells were pretreated by rottlerin for 30 min and then challenged with 100 μg/ml poly(I:C) or PBS for 2 h. The cells were lysed by addition of 1 x loading buffer and analyzed by SDS-PAGE followed by immunoblotting using antibodies against phospho-p65, p65, phospho-p38, p38, phospho-JNK, JNK, phospho-ERK, ERK, phospho-PKCδ, PKCδ, IκBα, A20, β-actin, phospho-hA20, Myc, and Flag. The IL-33 antibody was purchased from R&D Company and other antibody reagents were obtained from Cell Signaling Technology. Quantification of Western blots was performed with ImageJ software.

### Immunofluorescent Staining

BMDM cells were washed three times with PBS, fixed on coverslips with 2.5% paraformaldehyde (Sigma) for 10 min at room temperature, rinsed twice with PBS, and treated with 0.5% Triton X-100 (Roche Molecular Biochemicals, Indianapolis, IN, United States) for 10 min at room temperature. Cells were then blocked with 100 μl of 3% BSA for 1 h followed by addition of 100 μl of NF-κB antibody diluted 1:500 in 3% BSA overnight at 4°C, and then washed with PBS (3 × 5 min). The FITC-conjugated goat anti-rabbit antibody was diluted 1:1000 and 100 μl of the antibody solution was placed on each coverslip for 1 h at room temperature, followed by washing with PBS (3 × 5 min). Nuclei were stained with 0.1 μg/ml propidium iodide. The coverslips were mounted face down on microscope slides with mounting medium (Vector Laboratories, Inc., Burlingame, CA, United States) and viewed on a Zeiss 410 confocal microscope (Carl Zeiss, Germany). The slides were stored in a lightproof black box. The fluorescence intensity in the nucleus and cytoplasm were analyzed and calculated by Laser software.

### Cell Culture, Transfection, and Co-immunoprecipitation (Co-IP)

HEK-293T cells were obtained from ATCC and cultured in DMEM supplemented with 10% FBS. Raw264.7 cells were obtained from ATCC and grown in RPMI containing 10% FBS. Since commercial anti-mouse phosphorylated A20 antibodies are not available, we constructed eukaryotic expression plasmids for human PKCδ and human A20 which respectively been fused with Flag tag and Myc tag in order to detect their interaction. Flag-PKCδ and Myc-A20 were generated by PCR and were respectively cloned into the BamH1, Xbal1 and EcoR1, Xbal1 sites of pcDNA3.1. The full-length A20 protein was divided into three truncated regions, A20-1(1-383aa) included OTU domain, A20-2 (384-790aa) included Zn fingers domain and A20-3 (258-491aa) was an overlap region between A20-1 with A20-2. HEK-293T cells were transfected with pcDNA3.1-Flag-PKCδ, pcDNA3.1-Myc-A20 or three A20 truncates expression vectors by PEI method. We detected the phosphorylation level of human A20 in cells that were co-transfected with a different quantity of pcDNA3.1-Flag-PKCδ by WB. The co-IP assays were performed as previously described to detect the binding interaction between PKCδ and A20 both in 293T cells and macrophages (Yang et al., 2019). After addition of the appropriate amount of RIPA buffer (Beyotime, Shanghai, China) to the cell culture plate, and the lysate was transferred to a 1.5 ml EP tube then placed on ice for 15 min for full lysis. Lysates were centrifuged at 14,000 g for 15 min and the supernatant collected. A small amount of lysate was used for subsequent WB analysis. To remove non-specific protein binding, an appropriate amount of 50% protein G agarose was added to the remaining lysate and gently shaken for 10 min on the ice. The samples were then centrifuged at 3000 g for 30 s and the supernatant collected. The appropriate corresponding antibody (1 μg) was added to the supernatant, which was then gently shaken and incubated for 5 h at 4°C. Protein G agarose beads were washed three times with an appropriate amount of lysis buffer and centrifuged at 3000 g for 30 s before using. Pretreated protein G agarose beads were added to the cell lysate, which was incubated overnight at 4°C with gentle shaking and centrifuged at 3000 g for 30 s at 4°C. The supernatant was removed with a pipette and the agarose beads washed three times with 1 ml of lysis buffer. Then, an equal volume of 2 × SDS loading buffer was added to the beads which were incubated in a 100°C heating block for 5 min and subjected to SDS-PAGE and WB analysis.

### Statistical Analysis

All data are presented as means ± SEM. Differences between 2-experimental groups were analyzed using Student’s *t*-test. One-way ANOVA, followed by Dunnett’s *post hoc* test, was used for multiple comparisons. Prism 5.0 software (GraphPad Software, La Jolla, CA, United States) was used for statistical analyses. A value *P* < 0.05 was considered statistically significant.

## Results

### PKCδ Inhibits BLM-Induced Idiopathic Pulmonary Fibrosis

**FIGURE 1:**
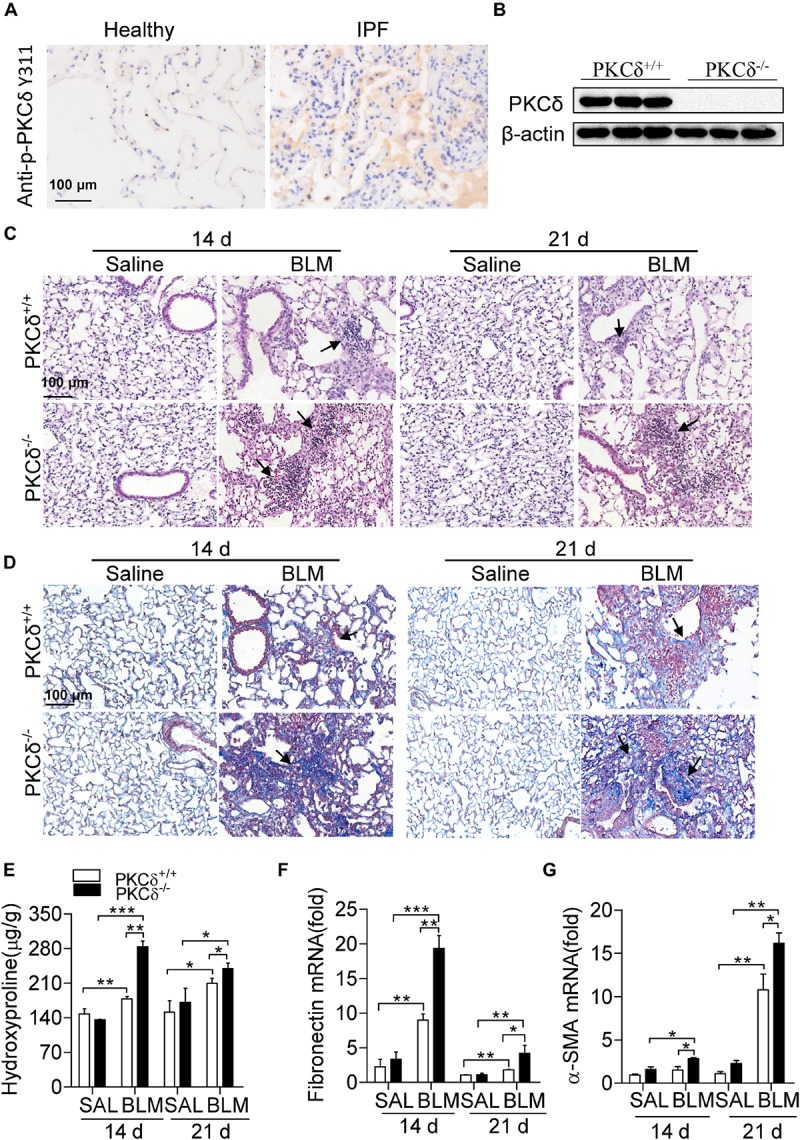
PKCδ deficiency enhances BLM-induced pulmonary fibrosis. **(A)** p-PKCδ staining by IHC in lung tissue of patients with IPF and that of healthy human (original magnification ×200). **(B)** To identify PKCδ knockout mice, we collected the lung tissues of PKCδ^+/+^ and PKCδ^–/–^ mice (*n* = 3) to detect the expression of PKCδ protein by western blotting. **(C)** PKCδ^+/+^ and PKCδ^–/–^ mice were injected intratracheally with saline or BLM (1.6 U/kg) (*n* = 5–8 mice in each group), after 14 and 21 days lung samples were collected, sectioned and stained with H&E (original magnification ×400), the arrows indicate infiltration of inflammatory cells. **(D)** Masson trichrome staining was performed to detect collagen deposition in the lung tissue of PKCδ^+/+^ and PKCδ^–/–^ mice, treated as described in **(C)** (original magnification ×400), and the arrows indicate collagen deposition. **(E)** Hydroxyproline was detected in the lung tissue of mice, treated as described in **(C)**. The expression of fibronection **(F)** and α-SMA **(G)** was detected by quantitative RT-PCR in samples from the lung tissue of mice, treated as described in **(C)**. *p < 0.05, **p < 0.01, ***p < 0.001.

To investigate whether the activation of PKCδ plays a role in the pathogenesis of pulmonary fibrosis in human, we detected the PKCδ phosphorylation in the lung tissue of patients with pulmonary fibrosis and that of healthy human by immunohistochemistry (IHC) staining. As shown in Figure 1A, the PKCδ phosphorylation in the lung tissue of patients was significantly higher than that of healthy human. These results indicated that PKCδ activation is involved in human pulmonary fibrosis. To determine whether PKCδ modulates IPF, we examined the effect of PKCδ on BLM-induced pulmonary fibrosis by using PKCδ deficient mice. As shown in Figure 1B, the expression of PKCδ was ablated in the lung tissue of PKCδ^–/–^ mice. Fourteen and twenty one days after BLM treatment, lung tissue of PKCδ^–/–^ mice displayed more aggravated multifocal fibrotic pulmonary lesions and inflammatory cell accumulation (Figure 1C). By using Masson trichrome staining, we found that the pulmonary interstitium of PKCδ^–/–^ mice contained more collagen deposition than that of PKCδ^+/+^ mice (Figure 1D). In addition, the expression of hydroxyproline (Figure 1E), fibronectin (Figure 1F), and alpha smooth muscle actin (α-SMA) (Figure 1G) was up-regulated in the lung tissue of PKCδ^–/–^ mice after BLM treatment, compared to that of PKCδ^+/+^ mice. Collectively, these data suggested that PKCδ inhibits BLM-induced pulmonary fibrosis.

### PKCδ Attenuates BLM-Induced Pulmonary Inflammation

**FIGURE 2:**
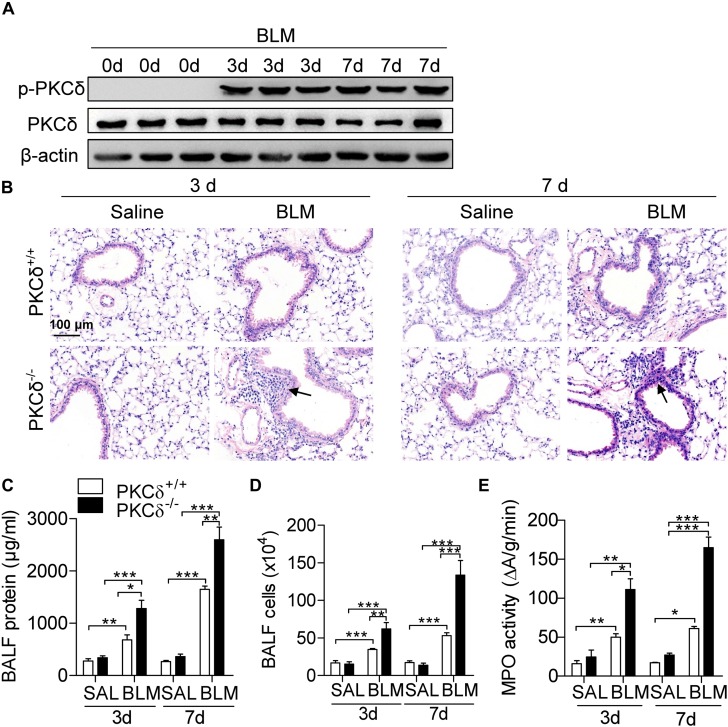
PKCδ deficiency enhances BLM-induced inflammation. **(A)** PKCδ^+/+^ mice were injected intratracheally with BLM (1.6 U/kg) (*n* = 3) for 0, 3, and 7 days. Collected the lung tissues to detect the phosphorylation of PKCδ by western blotting. **(B)** PKCδ^+/+^ mice and PKCδ^–/–^ mice were intratracheally injected with saline or BLM (*n* = 5–8 mice in each group), after 3 days and 7 days, lung sections were harvested and stained with H&E (original magnification × 400), the arrows indicate infiltration of inflammatory cells. The total protein concentration **(C)** and the total number of infiltrating cells **(D)** in BALF from mice treated as described in **(B)**, were quantified. **(E)** The level of MPO activity was detected in lung tissue of the mice, as described in **(B)**. *p < 0.05 **p < 0.01, ***p < 0.001.

Given inflammation is important in the development of pulmonary fibrosis, we determine whether PKCδ regulates BLM-induced pulmonary inflammation. As shown in Figure 2A, the PKCδ phosphorylation (Phospho-Tyr311) in lung tissue was obviously increased by BLM treatment for 3 and 7 days. The lung tissue of PKCδ^–/–^ mice displayed more aggravated lung injury and inflammatory cell infiltration than that of PKCδ^+/+^ mice (Figure 2B). In addition, the total protein concentration (Figure 2C) and inflammatory cells (Figure 2D) in BALF and the activity of myeloperoxidase (MPO) (Figure 2E) in the lung tissue were dramatically increased in PKCδ^–/–^ mice after BLM treatment. These data indicated that PKCδ inhibits BLM-induced pulmonary inflammation.

### PKCδ Alleviates BLM-Induced Cytokine Production in the Lung Tissue

**FIGURE 3:**
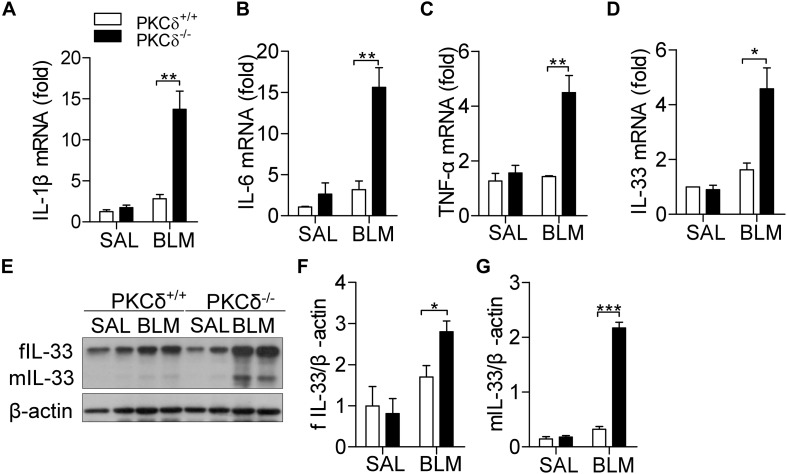
PKCδ deficiency increases cytokine production in the lung tissue of a BLM-induced mice model. PKCδ^+/+^ mice and PKCδ^–/–^ mice were challenged by saline or BLM (1.6 U/kg). After 3 days, the mRNA expression of IL-1β **(A)**, IL-6 **(B)**, TNF-α **(C)**, and IL-33 **(D)** in lung tissues was detected by qPCR. **(E)** Full length IL-33 (fIL-33) and mature IL-33 (mIL-33) protein expression was detected by western blotting in lung tissues of mice treated as described in **(A)**. The statistical results of fIL-33 **(F)** and mIL-33 **(G)** were analyzed by ImageJ software. *p < 0.05 **p < 0.01, ***p < 0.001.

Inflammatory cytokines play a critical role in the regulation of IPF (Hallstrand et al., 2014). Three days after BLM treatment, the production of IL-1β (Figure 3A), IL-6 (Figure 3B), TNF-α (Figure 3C), and IL-33 (Figure 3D) were up-regulated in the lung tissue of PKCδ^–/–^ mice, compared to that in PKCδ^+/+^ mice. As shown in Figures 3E–G, PKCδ deficiency enhanced the expression of both full-length and mature IL-33 protein in lung tissue, in which mature IL-33 has biological function. These data suggested that PKCδ inhibits the expression of inflammatory cytokines in *in vivo*.

### PKCδ Attenuates Cytokine Production in Macrophages

**FIGURE 4:**
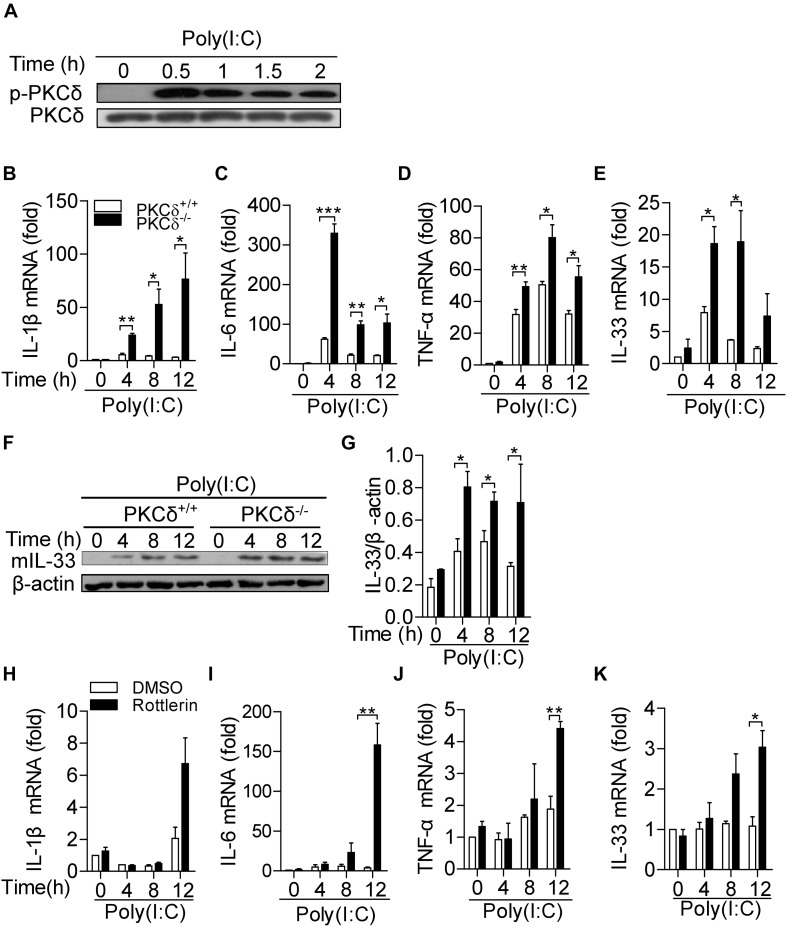
PKCδ attenuates cytokine production in macrophages. **(A)** The BMDMs from PKCδ^+/+^ mice were stimulated by addition of 100 μg/ml poly(I:C) for 0, 0.5, 1.0,1.5, and 2 h. Cell lysate samples were collected to detect the phosphorylation level of PKCδ. BMDMs of PKCδ^+/+^ mice and PKCδ^–/–^ mice were stimulated by 100 μg/ml poly (I:C) for 0, 4, 8, and 12 h. After cell lysis, RNA samples were used to detect the expression levels of mRNA for IL1-β **(B)**, IL-6 **(C)**, TNF-α **(D),** and IL-33 **(E)**. **(F)** Mature IL-33 (mIL-33) protein expression was detected by WB in the lysates of poly(I:C) stimulated BMDM, as described in **(E)**. **(F)** The statistical results of mIL-33 were analyzed by ImageJ software. The Raw264.7 cells were pretreated by rottlerin (4 μm) or DMSO for 30 min and then stimulated by poly(I:C) for 0, 4, 8, and 12 h. After cell lysis, RNA samples were used to detect the levels of mRNA for IL-1β **(H)**, IL-6 **(I)**, TNF-α **(J)**, and IL-33 **(K)**. *p < 0.05 **p < 0.01, ***p < 0.001.

Given macrophages are critical immune cells in regulation of inflammatory response, we examined the effect of PKCδ on cytokine production in macrophages. As shown in Figure 4A, the PKCδ phosphorylation (Phospho-Tyr311) in macrophages was increased by poly(I:C) stimulation. After poly(I:C) treatment for 0, 4, 8, and 12 h, bone marrow derived macrophages (BMDMs) isolated from PKCδ^–/–^ mice produced more IL-1β (Figure 4B), IL-6 (Figure 4C), TNF-α (Figure 4D), and IL-33 (Figure 4E). Consistently, PKCδ also inhibits mature IL-33 protein expression in BMDM (Figures 4F,G) *in vitro*. In addition, to further demonstrate that PKCδ activation inhibits the inflammatory cytokines expression, rotterlin (4 μm) was used to inhibit the PKCδ activity in poly(I:C) stimulated Raw264.7 cells (Arisaka et al., 2010) and the expression of IL-1β, IL-6, TNF-α, and IL-33 was detected. The results showed that rottlerin could significantly enhance the expression of IL-6 (Figure 4I), TNF-α (Figure 4J), and IL-33 (Figure 4K) but not the expression of IL-1β (Figure 4H) in the poly(I:C) stimulated Raw264.7 cells. Collectively, these data suggested that PKCδ activation attenuates cytokine production in macrophages.

### PKCδ Inhibits NF-κB Signaling Activity and Increases A20 Expression

**FIGURE 5:**
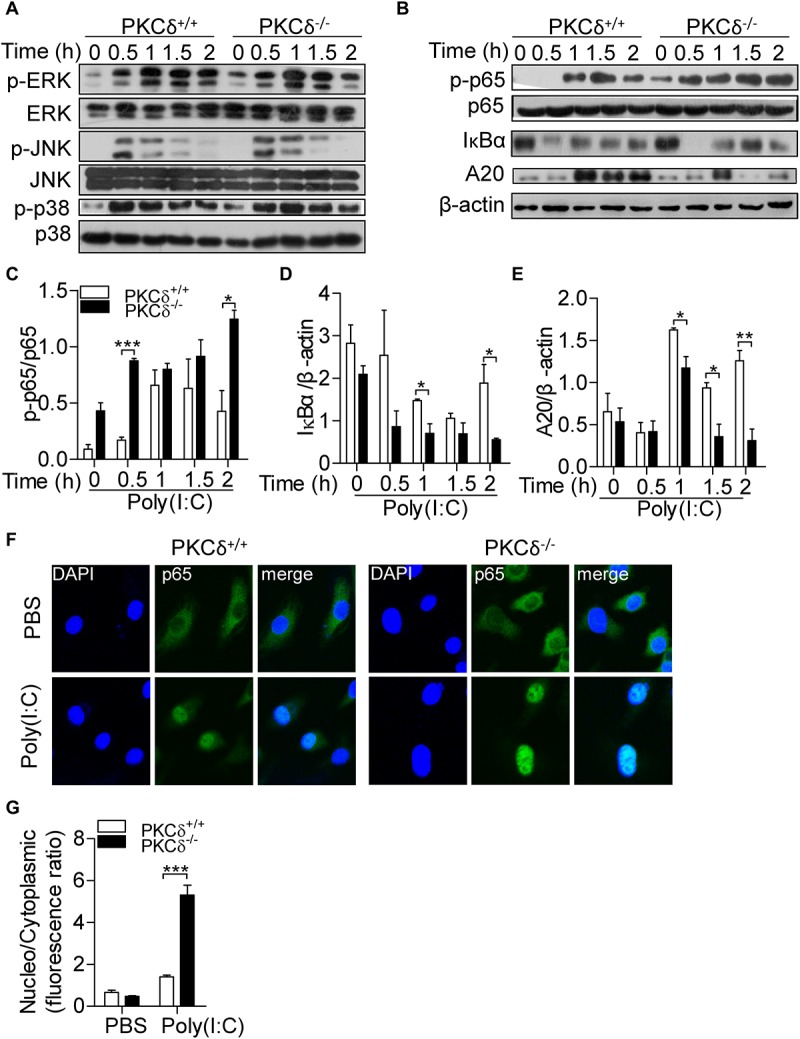
PKCδ deficiency enhances phosphorylation of p65 and expression of A20. **(A)** BMDMs from PKCδ^+/+^ mice and PKCδ^–/–^ mice were stimulated by addition of 100 μg/ml poly(I:C) for 0, 0.5, 1.0, 1.5, and 2 h, cells were lysed and used to detect the phosphorylation of MAPKs, including the ERK, JNK, and p38 MAPK. **(B)** Phosphorylation of p65, degradation of IκBα, and A20 expression were measured. The quantitative assay of p-p65 **(C)**, IκBα **(D)**, and A20 **(E)** were performed by ImageJ software. **(F)** Immunofluorescent staining was performed to detect the subceullar localization of p65 in the PKCδ^+/+^ and PKCδ^–/–^ BMDMs after poly(I:C) stimulation for 2 h. **(G)** The statistical results of the fluorescence ratio between the nucleus and the cytoplasm in BMDMs was analyzed by LAS AF Lite software. *p < 0.05 **p < 0.01, ***p < 0.001.

Among TLR3-mediated macrophage activation, MAPK and NF-κB signals are required for cytokine production including IL-33 (Li et al., 2017). To determine how PKCδ regulates cytokine production, we examined the effect of PKCδ on the activity of MAPK and NF-κB signaling pathways. As shown in Figure 5A, PKCδ had no significant effect on the phosphorylation of JNK, ERK, and p38 MAPK. However, in response to poly(I:C) treatment, the phosphorylation of p65 and degradation of IκBα were increased in PKCδ deficient BMDMs (Figures 5B–D; Straughn et al., 2019). In addition, the expression of A20 was attenuated in PKCδ deficient BMDMs (Figures 5B,E). To further confirm the role of PKCδ in NF-κB activation, immunofluorescent microscopy was performed to define the subcellular localization of the p65 in poly(I:C) stimulated-BMDM. As shown in Figures 5F,G, the transport of p65 into the nucleus was increased in PKCδ deficient macrophages. These data indicated that PKCδ inhibits NF-κB signaling activity and enhances the expression of A20.

### PKCδ Directly Binds to and Phosphorylates A20

**FIGURE 6:**
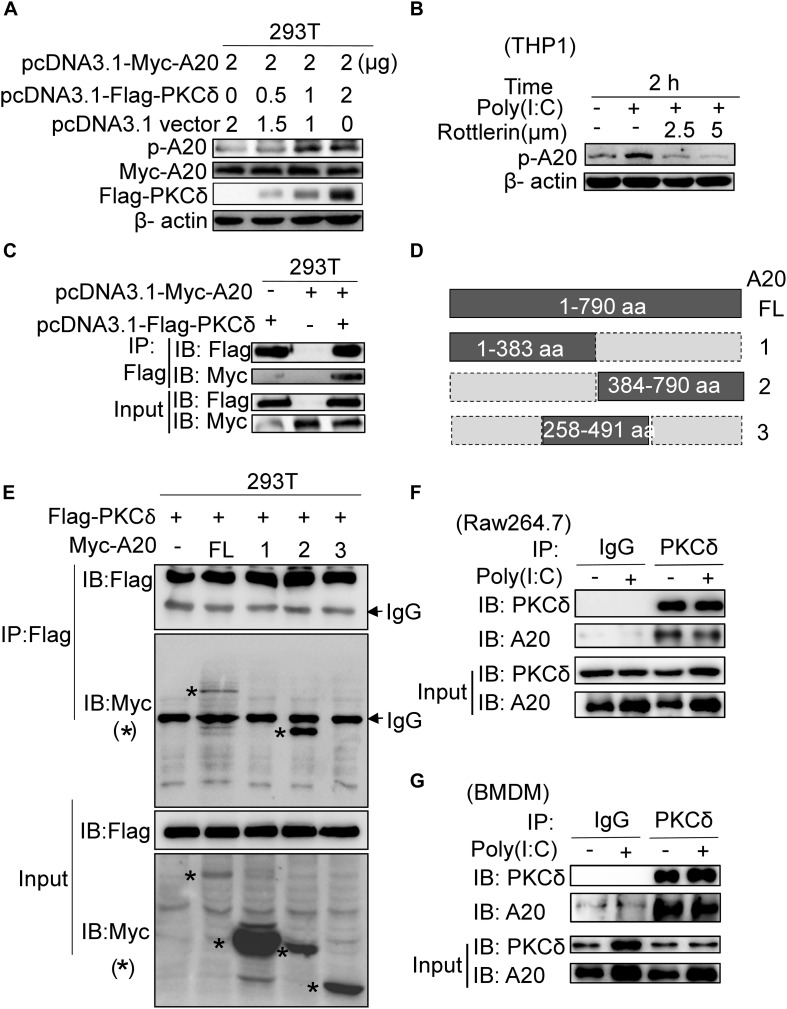
PKCδ directly binds and phosphorylates A20. **(A)** 293T cells were transfected with 0, 0.5, 1, 2 μg pcDNA3.1-Flag-PKCδ respectively and 2 μg pcDNA3.1-Myc-A20. Forty eight after transfection, the expression of Myc-A20, Flag-PKCδ, and the phosphorylation of A20 was detected by WB. **(B)** THP1 cells were pretreated by 2.5 or 5 μm rottlerin for 30 min and then stimulated by poly(I:C) for 2 h. The endogenous A20 phosphorylation was detected by WB. **(C)** Co-IPs were performed to detect the binding interaction between human PKCδ and A20 in 293T cells by using anti-Flag antibody to immune-precipitate cell lysate. **(D)** The schematic diagram of full length A20 and three truncated regions. **(E)** Co-IPs were performed to detect the region that human PKCδ binds to A20. **(F)** Raw264.7 cells were treated with poly(I:C) or PBS for 2 h. Cell lysate was immunoprecipitated with anti-PKCδ antibody, co-IP was performed to detect the interaction of endogenous PKCδ and A20. **(G)** BMDM cells were also treated with poly(I:C) or PBS for 2 h. And cell lysate was immunoprecipitated with anti-PKCδ antibody, co-IP was performed to detect the interaction of endogenous PKCδ and A20. The “*” represent the specific bands corresponding to the target proteins.

According to that PKCδ deficiency is associated with decreased expression of A20, we next determined whether PKCδ can bind to and phosphorylate A20. As shown in Figure 6A, the phosphorylation of A20 was increased in HEK293 cells that were co-transfected with Flag-PKCδ and Myc-A20. Meanwhile, rottlerin was used to inhibit the endogenous PKCδ activation in the poly(I:C) stimulated THP1 cells and the A20 phosphorylation was detected. We found that both 2.5 μm and 5 μm concentrations of rottlerin could inhibit the A20 phosphorylation (Figure 6B). In addition, PKCδ directly bound to A20 based on immunoprecipitation assay (Figure 6C). As shown in Figures 6D,E, A20 was divided into three region (A20-1 1-383aa, A20-2 384-790aa, A20-3 258-491aa) and only A20-2 region was bound with PKCδ, suggesting that the 492-790aa region of A20 bound to PKCδ. Additionally, we carried out immunoprecipitation experiments by using anti-PKCδ and anti-A20 antibodies, the endogenous PKCδ directly bound to A20 in both Raw264.7 cells (Figure 6F) and BMDMs (Figure 6G) with no effect of poly(I:C). Therefore, these results suggested that PKCδ directly binds to and phosphorylates A20.

## Discussion

Idiopathic pulmonary fibrosis (IPF) is a chronic and fatal interstitial lung disease for which current therapies have limited efficacy (Dempsey, 2006). It is imperative to improve our understanding of IPF development for the invention of effective treatments. Although PKCδ has been reported to be involved in the progression of pulmonary fibrosis, the role of PKCδ in IPF is still under controversy. Here, we found that the PKCδ phosphorylation was significantly increased in lung tissue of patients with pulmonary fibrosis and the PKCδ deficiency enhanced BLM-induced inflammation and pulmonary fibrosis, suggesting that PKCδ plays a protective role in IPF. In addition, we identified that PKCδ could bind to and phosphorylate A20, which suggested that PKCδ may inhibit NF-κB signal via promoting the phosphorylation and stability of A20. Our findings revealed that PKCδ reduces IPF development by attenuating NF-κB signaling.

In this study, we found that PKCδ deficient mice displayed severe inflammation and pulmonary fibrosis in BLM-induced pulmonary fibrosis model, which was controversial in the field. PKCδ has been reported to enhance the expression of fibronectin, collagen, and α-SMA in fibroblasts, indirectly suggesting that PKCδ promotes IPF (Kucich et al., 2000; Jimenez et al., 2001; Nakanishi et al., 2015). However, most these studies used PKCδ inhibitor rottlerin to inhibit the activity of PKCδ, which might cause some non-specific inhibitory effects, such as on PKCα and PKCβ (Gschwendt et al., 1994; Jimenez et al., 2001; Nakanishi et al., 2015). In addition, PKCδ deficient mice protect against asbestos-induced pulmonary fibrosis via promoting proinflammatory and profibrotic cytokine expression (Shukla et al., 2007). The inconsistent results from PKCδ deficient mice may be caused by different stimulant conditions and mice strains. In our study, PKCδ deficient mice were used to investigate the role of PKCδ in pulmonary fibrosis in BLM-induced pulmonary fibrosis mouse model. Even though we used BLM-insensitive Balb/c background mice, PKCδ deficiency still obviously exacerbated inflammation and fibrosis, suggesting that PKCδ plays a negative role in the pathological process of IPF. Based on our results, PKCδ is an inhibitory kinase in macrophage activation and BLM-induced pulmonary fibrosis.

Macrophages, as a central contributor to pulmonary fibrosis, have many effects on regulating fibrotic response (Desai et al., 2018). However, the role of PKCδ in macrophage activation is unknown. In this study, PKCδ deficient macrophages generated more inflammatory cytokines, which was associated with enhanced pulmonary fibrosis, suggesting that PKCδ may inhibit pulmonary fibrosis through attenuation of inflammatory cytokine production. Among the various proinflammatory cytokines we detected, IL-33 is an important enhancer of the inflammatory response that could be induced in active macrophages. IL-33 plays critical roles in metabolic homeostasis, infection, inflammation, cancer and central nervous system diseases (Liew et al., 2016). The IL-33-mediated signal pathway is also required for pulmonary diseases (Xu et al., 2016; Gabryelska et al., 2019). In BLM-induced pulmonary fibrosis model, ST2 deficiency, anti–IL-33 antibody treatment, or alveolar macrophage depletion attenuated BLM-induced lung inflammation and fibrosis. Inversely, exogenous IL-33 or adoptive transfer of ILC2s enhanced BLM-mediated pulmonary fibrosis (Li et al., 2014). Therefore, IL-33 is a novel profibrogenic factor that promotes the initiation and progression of BLM-induced IPF by recruiting inflammatory cells and enhancing profibrogenic cytokine production in a macrophage-dependent manner (Li et al., 2014). The effect of PKCδ on regulating cytokine expression in macrophage is still controversial based on data from different groups. PKCδ inhibits IFN-γ-stimulated IL-6 expression in BMDMs (Anita Schwegmann et al., 2007) while promotes IL-1β and IL-6 expression in trehalose 6,6-dibehenate (TDB)-induced macrophages (Duan et al., 2018). Our results indicated that PKCδ inhibits poly(I:C)-triggered cytokines included IL-33 production in macrophages. Therefore, the effect of PKCδ on inflammatory cytokine production should be further investigated by using more stimulants and more inflammatory signal pathways should be detected.

Several inflammatory signals are involved in the regulation of proinflammatory cytokine expression (Polumuri et al., 2012). In macrophages, the NF-κB signal is involved in the pathogen-associated molecular patterns (Theoharides et al., 2015), while mitogen-activated protein kinase (MAPK) signaling is associated with RSV (Qi et al., 2017). In this study, we demonstrated that PKCδ inhibits the activity of NF-κB but not the MAPK pathway, which reduces the cytokine expression. Several groups found that PKCδ can regulate on NF-κB signal pathways (Onose et al., 2006; Bijli et al., 2008; Minhajuddin et al., 2009). For instance, PKCδ promotes thrombin-induced NF-κB activation by phosphorylation of protein-tyrosine kinase Syk in endothelial cells (Bijli et al., 2008). Activated PKCδ leads to activation of the IκBβ kinase that, in turn, phosphorylates IκBα and triggers NF-κB activation (Rahman et al., 2002). In this study, we found that PKCδ inhibited TLR3-mediated NF-κB activation in macrophage. As a serine threonine kinase, PKCδ regulates gene expression by phosphorylating other various substrate proteins in both the cytoplasm and the nucleus (Brodie and Blumberg, 2003). In addition to NF-κB, several other transcription factors have also been regulated by PKCδ, such as Sp1, p300, Stat1, Stat3, among others (Novotny-Diermayr et al., 2002; Yuan et al., 2002; Kim et al., 2007; Kwon et al., 2007). In this context, phosphorylation of the Sp1 transcription factor promotes cyclin D3 expression in cells treated with the histone deacetylase apicidin (Kim et al., 2007). PKCδ also phosphorylates the acetyl transferase p300 at Ser89 inhibiting its activity *in vitro* and *in vivo* (Yuan et al., 2002). Moreover, Stat1 is phosphorylated at Ser727 to allow the transcription of the CIITA promoter (Kwon et al., 2007) and Stat-3 phosphorylation enhances the interaction between Stat3 and IL-6 receptor subunit glycoprotein (gp) 130 (Novotny-Diermayr et al., 2002). Due to the fact that NF-κB is the most extensive signaling pathway for inflammatory response, our research focused on activating NF-κB in cytoplasm by enhancing A20 phosphorylation which is regulated by poly(I:C) stimulation. We firstly identified that A20 was a new substrate of PKCδ which could be bound- and phosphorylated- by PKCδ. Since A20 is an effective inhibitor of the NF-κB signaling pathway, phosphorylation of A20 enhances its stability and inhibitory activity, thereby reducing the activation of NF-κB pathway (Hutti et al., 2007). These results indicated that PKCδ plays a different role in the NF-κB pathway, which may depend on cell types and different stimulants. In TLR3-mediated macrophage activation, PKCδ is a negative feedback regulator to inhibit NF-κB pathway and balance the inflammatory response. Interestingly, A20 is not only an inhibitor of NF-κB but also its target gene, the temporal relationship between the stability of PKCδ enhanced A20 protein and the down-regulation of A20 expression by NF-κB pathway needs further investigation.

NF-κB activity is tightly controlled by different signals, one of which is an important regulatory zinc finger (de) ubiquitinating enzyme A20, which can be induced by tumor necrosis factor receptor (TNFR) and Toll-like receptor (TLR) pathways. Generally, A20 serves as a (de)ubiquitinating enzyme to deactivate the NF-κB signal pathway (Shembade et al., 2010; Shembade and Harhaj, 2012; Das et al., 2018). The A20 activity can be regulated by phosphorylation on several sites. The phosphorylation of A20 at Ser381 by IκB kinaseβ enhances downregulation of pro-inflammatory signaling (Hutti et al., 2007). Except for Ser381, Ser480, Ser565, and Thr625 are also important for A20 activity, alanine substitution of all four phosphorylated residues or of Ser381 alone attenuate cleavage of K63-linked tetraubiquitin (Wertz et al., 2015). Here, we detected that PKCδ could bind to A20 and also phosphorylate it at Ser381 that may enhance its stability and activity. Several inflammatory and autoimmune diseases are correlated with the increased expression of A20, such as polyarthritis, inflammatory bowel disease, cystic fibrosis, and chronic inflammatory lung disease (Kang et al., 2009; Catrysse et al., 2014; Bannon et al., 2015; Momtazi et al., 2019). Therefore, in this study, not only did we find that A20 is a new substrate that is bound to- and phosphorylated by PKCδ, but also confirmed that excessive expression of A20 inhibits pulmonary inflammation and finally reduces IPF.

Collectively, our study defined an important role for PKCδ in BLM-induced pulmonary fibrosis and inflammation. We demonstrated that PKCδ inhibits the activation of the NF-κB signal pathway by binding and phosphorylating A20, which in turn reduces the expression of IL-33 and alleviates IPF, suggesting that PKCδ is a potential drug target for treating IPF.

## Data Availability Statement

All datasets generated for this study are included in the article/supplementary material.

## Ethics Statement

The animal study was reviewed and approved by the Institutional Animal Care and Use Committee at Shanghai Jiao Tong University.

## Author Contributions

JW, YN, SD, TZ, and WW performed the experiments. JW and LS analyzed the data and wrote the manuscript. JW and FQ designed the study. RY, SH, and FQ provided critical review of the manuscript.

## Conflict of Interest

The authors declare that the research was conducted in the absence of any commercial or financial relationships that could be construed as a potential conflict of interest.
